# Tomato Transcription Factor *SlWUS* Plays an Important Role in Tomato Flower and Locule Development

**DOI:** 10.3389/fpls.2017.00457

**Published:** 2017-03-31

**Authors:** Hui Li, Mingfang Qi, Meihua Sun, Ying Liu, Yudong Liu, Tao Xu, Yanbing Li, Tianlai Li

**Affiliations:** ^1^Key Laboratory of Protected Horticulture, Ministry of Education, Shenyang Agricultural UniversityShenyang, China; ^2^Collaborative Innovation Center of Protected Vegetable Surround Bohai Gulf RegionShenyang, China; ^3^Liaoyang Academy of Agricultural and Forestry ScienceLiaoyang, China

**Keywords:** *SlWUS*, flower organogenesis, fruit size, locule number, tomato

## Abstract

Tomato is a model species for fleshy fruit development. The shapes and sizes of tomato (*Solanum lycopersicum L.*) are mainly controlled by several loci, including locule number (*lc*). Two single nucleotide polymorphisms were found downstream of *WUSCHEL* (*SlWUS*) in a putative tomato CArG *cis*-regulatory element. The *lc* mutation may affect the binding of *AGAMOUS*(*AG*), cause the up-regulation of *SlWUS* and result in increased locule numbers. In this study, tissue expression levels showed that *SlWUS* is expressed in young floral buds and shoot apexes. Silencing *SlWUS* on an *lc* mutant genetic background with an RNA interference (RNAi) strategy resulted in smaller flowers and fruit than those of the wild-type plants, with decreased locule number. Further study revealed that the *SlWUS* RNAi lines exhibited altered expression levels of the *TAG1* and *SlCLV3* genes that participate in the regulation of tomato flower and fruit locule development. In conclusion, this study provides the first genetic evidence that *SlWUS* may be the candidate gene of the *lc* locus and reveals the function of *SlWUS* in flower development.

## Introduction

Tomato (*Solanum lycopersicum L.*) is an ideal model plant for fruit development research (Klee and Giovannoni, 2011; Consortium, 2012). Fruit size is the primary characteristic of commercial tomato varieties and an important goal for tomato domestication. Domesticated tomato fruit is enlarged 1000 times compared to its wild progenitors, which is an extreme case. In this dramatic transition, both carpel cell division and carpel number determine the final size of tomato fruit (Tanksley, 2004). A relatively small number of genes are involved in these two processes. In the first process, negative regulation of *FRUIT WEIGHT 2.2* (*FW2.2*) (Frary et al., 2000), which located in the plasma membrane, is required for the control of carpel cell number (Liu and Tanksley, 2003). Hence, low expression levels of the large-fruited allele of *FW2.2* stimulate cell division, which leads to increases in the final size of tomato fruit. Nonetheless, increased locule number has the greatest effect on tomato fruit size, which is derived from the carpels in tomato flowers. Almost all wild tomatoes and several small-fruited tomato cultivars produce fruits with only two to four locules. However, most cultivars consumed today produce fruits with six or more locules (Tanksley, 2004). Therefore, increased locule number contributes as much as 50% variance to fruit enlargement and is believed to represent a late step in the substantial increase in tomato fruit size during domestication (Lippman and Tanksley, 2001; Tanksley, 2004). An increase in locule number is highly associated with an increase in the number of floral organs, especially the carpel, and this trait is controlled by multiple quantitative trait loci (QTL) (Barrero and Tanksley, 2004), and a few of these loci have been identified (Grandillo et al., 1999; Fernández-Lozano et al., 2015; Illa-Berenguer et al., 2015). To date, the locule number has been shown to be controlled by two loci-*fascinated* (*fas*) and *locule number* (*lc*) (Lippman and Tanksley, 2001; Knaap and Tanksley, 2003; Barrero et al., 2006). Significant epistatic interactions have been detected between *lc* and *fas* (Lippman and Tanksley, 2001; Barrero and Tanksley, 2004). The positive epistatic interaction between *lc* and *fas* suggests that they are part of a single regulatory network that controls the size of floral meristems (van der Knaap et al., 2014; Swinnen et al., 2016).

The mutation in *fas* results in increased carpel numbers with more significant effects on carpel number than *lc* (Lippman and Tanksley, 2001). The *fas* mutant phenotype was previously reported to be caused by loss of expression of a YABBY-like transcription factor (Cong et al., 2008). However, recent studies that were carried out to further dissect the contribution of the *fas* locus; these studies identified a 294-kb inversion on chromosome 11 with breakpoints in intron 1 of a YABBY-like gene and 1 kb upstream of the tomato *CLAVATA3* (*SlCLV3*), which demonstrated that a regulatory change in *SlCLV3* underlies the *fas* mutant phenotype (Huang and van der Knaap, 2011).

The *lc* locus regulates the number of locules, and *lc* mutation leads to a tomato fruit with more than the two to four locules (Barrero et al., 2006; Muños et al., 2011). The *lc* locus is located within a non-coding region, but the *lc* locus may correspond to two single nucleotide polymorphisms (SNPs), which are located 1,080 bp downstream of the tomato ortholog of *WUS* (Muños et al., 2011) that encodes a transcription factor that is essential to maintain stem cell identity in the shoot apical meristems (SAM) (Mayer et al., 1998; Clark, 2001). In Arabidopsis, increased expression of *WUS* results in increased floral organ number, which resembles the phenotype of the *lc* mutant (Mayer et al., 1998; Clark, 2001). Although the function of these two SNPs has not yet been identified, they may be involved in the regulation of the expression of *SlWUS* or other genes that play a major role in floral development (Muños et al., 2011).

The homeobox gene *WUSCHEL* (*WUS*) plays an important role in maintaining the balance between the proliferation and differentiation of stem cells in the meristems of *Arabidopsis thaliana*. *WUS* positively regulates the expression of *AGAMOUS* (*AG*), which is part of the MADS box transcription factor (Lenhard et al., 2001; Lohmann et al., 2001). *AG* plays a key role in regulating stamen and gynoecium determinacy (Yanofsky et al., 1990). Thus, WUS-induced expression of *AG* links meristem activities to organ identity processes. In reverse, *AG* down-regulates the expression of *WUS*, which provides the mechanism for promoting the cells at the center of the flower to differentiate into carpels (Lohmann et al., 2001; Liu et al., 2011). In Arabidopsis, *WUS* down-regulation is mediated by two downstream CArG *cis*-regulatory elements that bind to AG and lead to the epigenetic silencing of *WUS* (Tilly et al., 1998; Liu et al., 2011). Interestingly, the two SNPs located downstream of tomato *WUSCHEL* (*SlWUS*) are located in a putative tomato CArG *cis*-regulatory element (van der Knaap et al., 2014). It has been reported that *TAG1* silencing lines show defects in stamen and carpel development in tomatoes (Pan et al., 2010). Although floral determinacy in *Arabidopsis* relies on a negative autoregulatory mechanism involving *AG* and *WUS*, the interaction between *TAG1* and the *SlWUS* in tomato is unknown. Additional key components of the WUS signaling pathway are the CLAVATA (CLV) proteins (Schoof et al., 2000; Clark, 2001; Lenhard and Laux, 2003). Furthermore, the WUS and CLV3 feedback loop is closely connected to the control of meristem size in Arabidopsis (Schoof et al., 2000). The *fas* mutant phenotype is caused by a loss of expression of a *clv3*-like ortholog (*SlCLV3*) and changes in the regulation of *SlWUS* that underlie the *lc* locus (Muños et al., 2011).

The *lc* locus is reported to be a key determining factor of the final carpel number in tomato fruit. *SlWUS* has been proposed as a candidate gene for *lc*. To address the potential role of *SlWUS* in tomato locules, ribonucleic acid interference (RNAi) transgenic plants were produced, which lead to the down-regulation of the *SlWUS* gene in tomato. The flower and fruit phenotype in the *SlWUS* RNAi lines revealed the involvement of *SlWUS* in the control of tomato floral organs and fruit locule number. Further genetic analysis indicated that the expression levels of the *TAG1* and *SlCLV3* genes were altered in the *SlWUS* RNAi lines. These results suggested that *SlWUS* plays an important role in tomato flower and fruit development.

## Materials and Methods

### Plant Materials, DNA Extraction, and Marker Development for the *LC* Gene

Three tomato (*S. lycopersicum L.*) lines, ‘MLK1,’ ‘FL1,’ and ‘Zhongshu6’ were planted in May 2014 at Shenyang Agriculture University, Shenyang, China. The ‘MLK1’ fruit were large with 10 or more locules, the ‘FL1’ fruit were small with 2–4 locules and the ‘Zhongshu6’ fruit were large with 6–8 locules. The cultivated allele of *lc* is present in ‘MLK1’ and ‘Zhongshu 6,’ and the wild-type allele of *lc* is present in ‘FL1.’ With the exception of the differences in locule number, the sepals, petals, and other agronomic traits are similar among these three variants. Genomic DNA was isolated from the leaves of the above mentioned plants using a DNA Extraction Kit (Tiangen Biotech, Beijing, China) in accordance with the manufacturer’s instructions. The PCR-based markers developed by Rodríguez et al. (2011) were used to identify the recessive high-locule-number allele at the *LC* locus used in this work. The primers lc-F1 (5′-GTCTCTTGGATGATGACTATTGCACTTT-3′) and lc-R1 (5′-AAAGTAGTACGAATTGTCCAATCAGTCAG-3′) were used to amplify the low-locule-number allele, while lc-F2 (5′-CTTTTCCTAAAAGATTTGGCATGAGGT-3′) and lc-R2 (5′-TCAGCGCCTCATTTTCTATAGTATTTGT-3′) were used to amplify the recessive high-locule-number allele. When the cultivated allele is present, lc-F1 and lc-R1 will amplify a band of 533 bp; when the wild-type allele is present, lc-F2 and lc-R2 will amplify a band of 395 bp. The two primers were used in the same PCR master mix following the method described by Rodríguez et al. (2011).

### Construction of RNAi and Overexpression Vectors and Tomato Transformation

The cDNA of *SlWUS* was amplified from ‘Zhongshu6’ by real-time polymerase chain reaction (RT–PCR) using specific primers from tomato (GenBank accession number AJ538329) (Muños et al., 2011). The RNAi-specific primers used for the *SlWUS* gene are as follows: SlWUS-for (5′-CAACGAGCGATCAGATAAGAATA-3′) and SlWUS-rev (5′-ATGGACACTGAACACCTGGATTA-3′). The amplified fragment was transferred into the pCR8/GW/TOPO entry vector by means of a TOPO reaction (Invitrogen). The nucleotide sequence was verified, and the fragment was then introduced to the silencing vector pB7GWIWG2 with an LR recombinant reaction. The constructs were then introduced into *Agrobacterium tumefaciens* LBA4404 by electroporation.

The seeds of *S. lycopersicum* ‘Zhongshu6’ were surface sterilized, sown on 1/2 MS culture medium and germinated with a 16 h day/8 h night regime at 25°C for approximately 6–8 days. Cotyledon explants were cut and preincubated for 2 day on KCMS in the dark at 25°C. After preculturing, the cotyledon explants were cultured with an *A. tumefaciens* LBA4404 suspension liquid for 4 min with lightly shaking. After infection, the explants were returned to the premedium for 2 days in the dark at 25°C. Then, the cotyledon explants were transferred to selection medium including MS salts, 3% sucrose, 7 g/L agar, 2 mg/L 6-BA, 0.2 mg/L IAA, 400 mg/L cephalosporin and 0.5 mg/L glufosinate-ammonium to induce shoot generation. The cotyledon explants that regenerated new shoots were then transferred to rooting culture medium, which included MS salt, 3% sucrose, 5 g/L agar, 400 mg/L cephalosporin and 0.05 mg/L NAA. The non-transgenic lines and transgenic plants were planted in a greenhouse.

### RNA Extraction and Gene Expression Analysis by qRT–PCR

Samples were collected from 10 to 15 tomato plants and immediately frozen in liquid nitrogen (Cong et al., 2008) (Supplementary Figure 1). Total RNA was extracted using an RNA extraction kit (Tiangen Biotech, Beijing, China) according to the manufacturer’s instructions. Total RNA was treated by DNase I to remove any genomic DNA contamination. The first-strand cDNA was reverse transcribed from 2 mg of total RNA using the Omniscript kit (Qiagen) according to the manufacturer’s instructions. The real-time PCR analysis was performed as described by Jain et al. (2006). The cDNA samples were used as the template and mixed with 200 nmol of each primer and the SYBR Green PCR Master Mix (Tiangen, Beijing, China) for real-time PCR analysis using an ABI 7500 Real-Time PCR System and Software 7500 ver. 2.0.3 (Applied Biosystems, USA) according to the manufacturer’s instructions. The temperature procedure was: 95°C for 15 min, 40 cycles of 95°C for 30 s, 57°C for 30 s, and 70°C for 1 min. The fluorescence signal was measured during the extension at 70°C of each cycle. The *Ubiquitin3* gene from tomato was used as the control for normalization. The sequences of all primers that were used in this study are listed in Supplementary Table 1.

All values are expressed as the mean ± standard deviation of three independent experiments, and the data were analyzed using Origin 8.0. Analysis of variance was performed using one-way ANOVA with SPSS 13.0 software followed by Duncan’s multiple range tests for each experiment at *P* < 0.05.

### Phenotypic Analysis of Tomato Flowers and Fruit

For flowers at the anthesis stage, 20 measurements (2 samples × 10 plants) of the widest diameter were calculated via a caliper gauge, and the sepal and petal areas were calculated. The number of sepals, petals, stamens, and carpels of the non-transgenic lines and transgenic plants was evaluated in tripped flowers. In addition, five mature fruits of the non-transgenic lines and transgenic plants were used to calculate the mean fruit weight (g), fruit width (mm), fruit length (mm), and the number of locules. All values are expressed as the mean ± standard deviation. The data were further subjected to analysis of variance, and least significant difference test was used to compare the average values. A probability of *P* < 0.05 was considered statistically significant.

## Results

### *SlWUS* Shows High Transcript Accumulation during Tomato Flower Development

**FIGURE 1:**
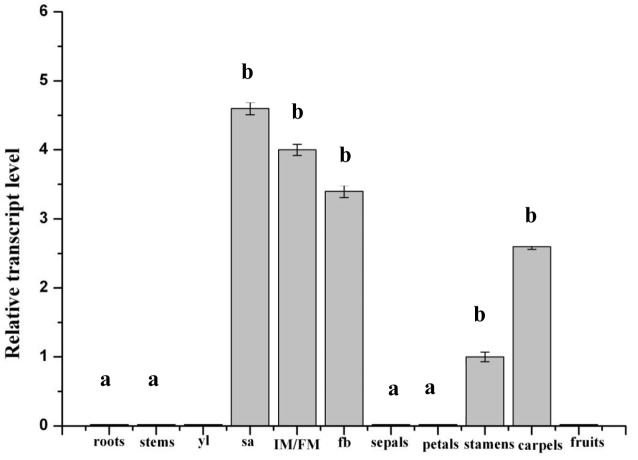
**Profiling of *SlWUS* transcript accumulation in wild-type tomato plant tissues monitored by qRT-PCR.** Samples were collected from the *Solanum pimpinellifolium* ‘Zhongshu 6.’ The level for stamens was used as a reference. yl, young leaves; sa, shoot apexes; IM/FM, infloresence meristem/floral meristem; yfb, young floral buds. The data are the mean values corresponding to three independent experiments. The error bars represent the standard errors. Values followed by the same letter (a or b) are not significantly different (*P* > 0.05, Duncan’s multiple range test).

Previous studies have reported that *SlWUS* is expressed in rapidly growing organs (Laux et al., 1996; van der Knaap et al., 2014). To more explicitly characterize this pattern of expression, RT-PCR analyses were carried out using *SlWUS* gene-specific primers. *SlWUS* expression was detected in the young flower buds, shoot apexes, and inflorescence meristem/floral meristem (IM/FM); expression was undetectable in the roots, stems, leaves, and fruits. In the flowers, *SlWUS* transcripts were detected in the stamens and carpels; expression was undetectable in the sepals and petals (**Figure 1**). An increase in locule number is highly associated with an increase in the number of floral organs, especially the carpel. Our results showed that *SlWUS* was expressed during flower development, which indicates an important function for *SlWUS* in the development of tomato flowers.

### Higher Expression of *SlWUS* Is Found in Plants on an *lc* Mutation Background

**FIGURE 2:**
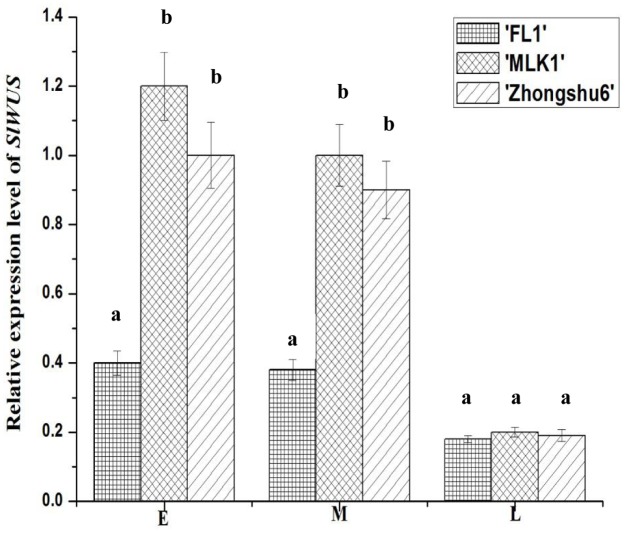
**Expression analysis of *SlWUS* in ‘FL1,’ ‘MLK1,’ and ‘Zhongshu6’ plants assessed by real-time PCR.** E, early development before the initiation of carpel primordial; M, mid-stage development shortly after carpel initiation; L, late-stage development; ‘MLK1’ and ‘Zhongshu6,’ the mutant alleles of *lc*; ‘FL1,’ the wild-type alleles of *lc*. The data are presented as the mean (±SE) values corresponding to three independent experiments. Values followed by the same letter (a or b) are not significantly different (*P* > 0.05).

To elucidate the effect of the *lc* locus on the expression of *SlWUS*, we examined the expression of *SlWUS* in the flower buds of three tomato lines, including ‘MLK1,’ ‘FL1,’ and ‘Zhongshu6’. The ‘MLK1’ fruit were large with 10 or more locules, the ‘FL1’ fruit were small with 2–4 locules and the ‘Zhongshu6’ fruits were large with 6–8 locules. The cultivated allele of *lc* is present in ‘MLK1’ and ‘Zhongshu 6,’ and the wild-type allele of *lc* is present in ‘FL1.’ The results showed that *SlWUS* abundance decreased gradually during flower bud development. Meanwhile, at each developmental stage of the flower buds, the expression levels of *SlWUS* were higher in ‘MLK1’ and ‘Zhongshu6’ than those in ‘FL1’ (**Figure 2**), which indicated that the expression of *SlWUS* was the difference between the wild-type and mutant alleles of *lc*.

### The Silencing of *SlWUS* Affects Tomato Flower Development and Decreases Tomato Fruit Locules

**FIGURE 3:**
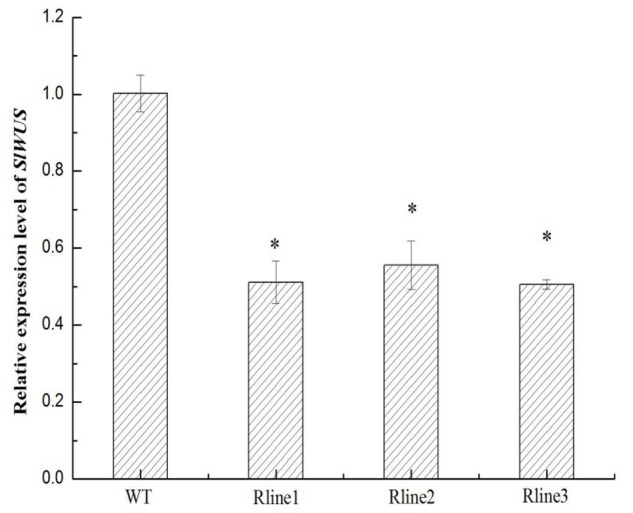
**Characterization of three independent *SlWUS* RNAi lines.** Transcript levels of *SlWUS* in young flower buds of three independent *SlWUS* RNAi lines (Rline1, Rline2, and Rline3) relative to those of wild type (WT). The data are presented as the mean (±SE) values corresponding to three independent experiments. ^∗^Indicates significant differences between transgenic lines and WT plants at *P* < 0.05, according to Duncan’s multiple range test.

**FIGURE 4:**
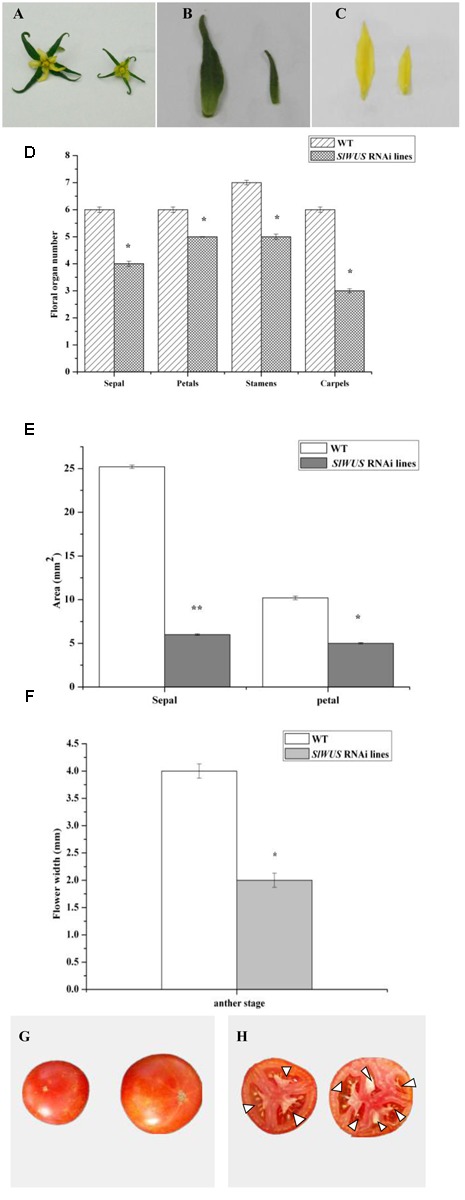
**Alteration of flower and fruit morphometrical characteristics in *SlWUS* RNAi plants. (A)** WT (left) and *SlWUS* RNAi plant flowers at anthesis. **(B)** WT (left) and *SlWUS* RNAi plants sepals at anthesis. **(C)** WT (left) and *SlWUS* RNAi plants petals at anthesis. **(D)** Mean comparison of the organ number in the four floral whorls at the anthesis stage. **(E)** Quantification of sepal and petal area in the control and *SlWUS* RNAi plant flowers at anthesis. The bars represent the standard error (*n* ≥ 100). **(F)** Mean comparison of flower width at the anthesis stage. **(G)** WT (right) and *SlWUS* RNAi plant mature fruit. **(H)** WT (right) and *SlWUS* RNAi plant fruit locule number. The data are presented as the mean (±SE) values corresponding to three independent experiments. ^∗^Indicates significant differences between transgenic lines and WT plants at *P* < 0.05, according to Duncan’s multiple range test. ^∗∗^Indicates significant differences between transgenic lines and WT plants at *P* < 0.01.

To further understand the function of *SlWUS* in tomato, we used a gene-specific region from the 3′ end of the cDNA (see “Materials and Methods”) to construct an RNA interference (RNAi) vector. The construct was introduced into Zhongshu6 by Agrobacterium-mediated transformation. There independent homozygous transgenic plants were produced for the construct. The gene expression analysis by real-time PCR showed that the expression levels of *SlWUS* were significantly reduced in the RNAi lines compared with those in the non-transgenic lines (**Figure 3**). All three of the *SlWUS* RNAi lines retained 50% of the control mRNA level. Therefore, one *SlWUS* RNAi line, Rline1, was selected for further study. The effects of *SlWUS* silencing on flower and fruit development were then investigated in the *SlWUS RNAi* lines. To characterize the flower development of the *SlWUS* RNAi lines, floral organ numbers at the anthesis stage were scored. The wild-type flowers were included in four whorls of floral organs and consisted of 5–6 sepals, 5–6 petals, approximately 6–7 stamens, and 6–8 carpels. In contrast, the *SlWUS RNAi* line flowers consisted of 4–5 sepals, 4–5 petals, 5–6 stamens, and 3–4 carpels. The number of carpels was significantly lower in the *SlWUS RNAi* lines than in WT (**Figures 4A,D**), which indicates that *SlWUS* gene function is essential to control carpel number during flower development. In addition, the width of the flowers were measured at the anthesis stage and revealed significant decreases in the *SlWUS RNAi* lines (**Figure 4F**). In addition, the *SlWUS RNAi* line flowers showed narrower sepals and petals, with remarkably decreased lamina area (**Figures 4B,C,E**). Therefore, the *SlWUS RNAi* lines differed significantly from WT with respect to the flower anthesis stage.

An increase in locule number is highly associated with an increase in the number of carpels in tomato. We observed that the wild-type plants yielded fruit with 6–8 locules, while the *SlWUS* RNAi lines yielded fruit with 3–4 locules (**Figure 4H**). Together with this decrease in locule number, there was a significant decrease in the weight and size of the fruit of the *SlWUS* RNAi lines (**Figure 4G** and **Table 1**). Altogether, the results indicated that the final size of the fruit of the *SlWUS* RNAi lines is determined by the decrease in the number of carpels that occurs during floral development.

**Table 1:** Comparison of mature fruits among the wild-type (WT) and *SlWUS* RNAi plants.

	Weight (g)	Number of locules	Fruit size	
			Width (mm)	Length (mm)
WT	174.9 ± 8.7^a^	6.7 ± 0.7^a^	86.7 ± 3.5^a^	72.8 ± 1.4^a^
*SlWUS* RNAi lines	85.9 ± 3.2^b^	3.3 ± 0.3^b^	63.0 ± 1.7^b^	58 ± 1.2^b^


*Values followed by the same letter (a or b) are not significantly different (*P* > 0.05, Duncan’s multiple range test)*.

### Expression Levels of a Carpel Development Gene Are Altered in the *SlWUS* RNAi Lines

**FIGURE 5:**
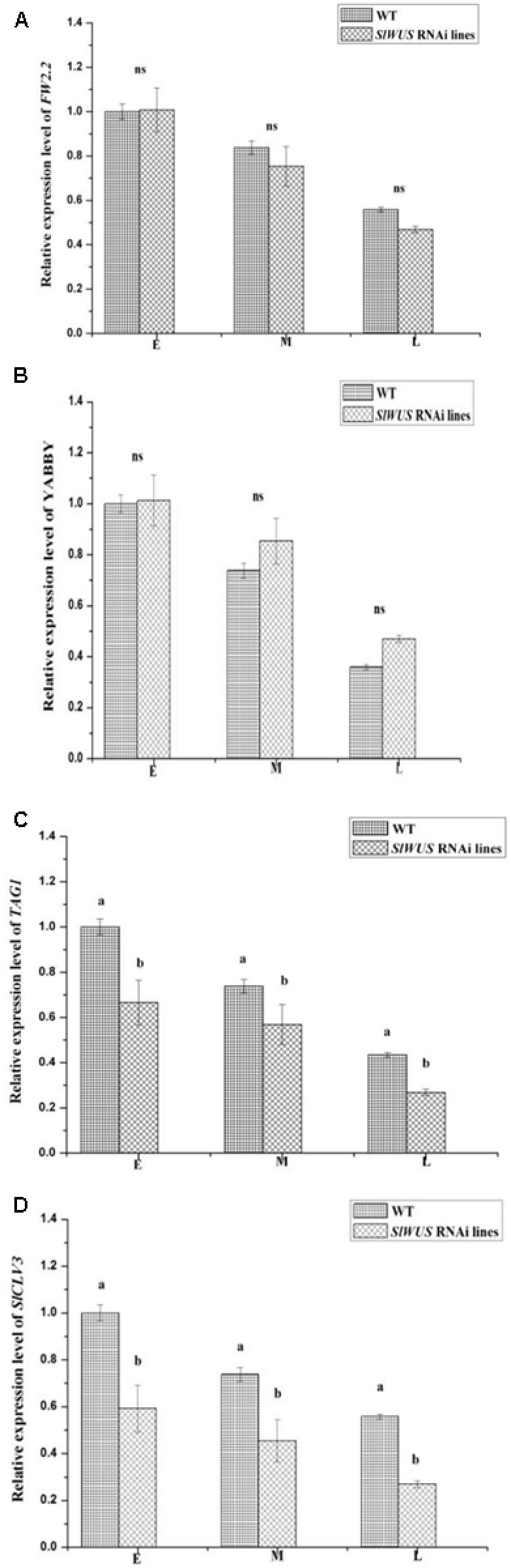
**Expression analysis of (A)** *FW2.2*, **(B)** *YABBY*, **(C)** *TAG1*, and **(D)** *SlCLV3* during flower development. E, early development before the initiation of carpel primordial; M, mid-stage development shortly after carpel initiation; and L, late-stage development. The data are presented as the mean (±SE) values corresponding to three independent experiments. ns, no statistically significant differences. Values followed by the same letter (a or b) are not significantly different (*P* > 0.05).

To further illustrate the mechanisms that are involved in controlling the flower and fruit locule number phenotypes observed in the *SlWUS* RNAi lines, the expression levels of the genes involved in the control of floral organ number and fruit size that were modified in the transgenic lines were analyzed. Thus, the transcript levels of *YABBY*, *TAG1*, *SlCLV3*, and *FW2.2*, genes were evaluated in the WT and *SlWUS* RNAi lines during flower development. The results showed that the expression levels of the *FW2.2* gene, a negative regulatory factor associated with carpel cell number (Frary et al., 2000), were not significantly different among the WT and *SlWUS* RNAi lines during flower development (**Figure 5A**). In addition, similar levels of *YABBY* gene expression were observed in the WT and *SlWUS* RNAi lines during flower development (**Figure 5B**). The expression levels of *TAG1* were significantly (*P* < 0.05) down-regulated in the *SlWUS* RNAi lines (**Figure 5C**). Similarly, the expression levels of *SlCLV3* were significantly (*P* < 0.05) down-regulated in the *SlWUS* RNAi lines during flower development (**Figure 5D**).

## Discussion

In tomato fruit, locule (cavities that develop from carpels) number ranges from 2 to 10 or more. The locules develop directly from the carpels in the tomato flower. It is known that *lc* is one of the key determinants of the final locule number in fruit, and *lc* mutation leads to a tomato fruit with more than two and four locules (Barrero et al., 2006; Muños et al., 2011). The *lc* locus is a non-coding region, which is located 1,080 bp downstream of the presumptive tomato homolog of *WUS* (Muños et al., 2011) that encodes a transcription factor that is essential to maintain stem cell identity in SAM (Mayer et al., 1998; Clark, 2001). Increased expression of *WUS* in Arabidopsis results in increased floral organ number, which is similar to the phenotype of the *lc* mutant (Mayer et al., 1998; Clark, 2001). Therefore, based on its predicted function, *SlWUS* is the most likely candidate gene for the *lc* locus (Barrero et al., 2006; van der Knaap et al., 2014). *WUS* encodes a homeodomain transcription factor that plays an important role in maintaining the balance between the proliferation and differentiation of stem cells in the meristems of *A. thaliana* (Laux et al., 1996; Mayer et al., 1998; Clark, 2001). The analysis of *SlWUS* revealed a similar expression pattern to that of *WUS* in Arabidopsis, which was detected in the young floral buds, SAM and the IM/FM (**Figure 1**). Furthermore, the *SlWUS* transcripts were detected in the stamens and carpels (**Figure 1**), which suggest a putative role for *SlWUS* in development of stamens and carpels in tomato. In addition, the phenotypic analysis of the stamens and carpels from the *SlWUS* lines revealed that *SlWUS* plays a role in the number of stamens and carpels (**Figures 4D,H**).

The tomato *lc* allele is associated with two SNPs downstream of *SlWUS*. These two SNPs were responsible for the increase in locule number, and they might participate in the regulation of *SlWUS* expression (Muños et al., 2011). In this study, the expression of *SlWUS* was the underlying difference between the wild-type and mutant alleles of *lc*, and higher expression of *SlWUS* was detected in the *lc* mutation background plants (**Figure 2**). These results suggest that an *lc* mutation may permit elevated expression of *SlWUS*. The maintenance of larger stem cells leads to increased locule numbers. Down-regulating *SlWUS* expression in the tomato transgenic plants resulted in a decreased fruit size (**Figure 4H** and **Table 1**). Fruit size is the primary characteristic of commercial tomato varieties and an important goal for tomato domestication. It is known that locule number largely affects the final fruit size in tomato. This increased locule number contributes as much as 50% variance to fruit enlargement (Lippman and Tanksley, 2001; Tanksley, 2004). The character analysis of tomato fruits from the *SlWUS* lines revealed that the decrease in fruit size was related to a decreased locule number (**Figures 4D,H**).

During tomato fruit domestication, both carpel cell division and carpel number determine the final size of tomato fruit (Tanksley, 2004). A relatively small number of genes were involved in the two processes. The *FW2.2* gene is responsible for the first process (Frary et al., 2000; Cong et al., 2002). Tomato fruit size is quantitatively controlled, and several QTLs were identified in this process (Paterson et al., 1991; Grandillo et al., 1999). Among these loci, the *FW2.2* gene largely governs fruit size (Frary et al., 2000; Ariizumi et al., 2013). The *SlWUS* RNAi lines did not show altered expression of the *FW2.2* gene during floral development (**Figure 5A**), which is involved in the negative control of the carpel cell division that is associated with carpel cell number (Frary et al., 2000). The result indicated that the decreased tomato fruit size in the *SlWUS* RNAi lines was not a result of the cell division process regulated by *FW2.2.* Whereas, the *fas* gene is the main determinant of the floral organ number of the second process, and mutations in this gene are considered major contributors that result in the increase in fruit size in modern cultivated species by increasing the locule number from two to more than six (Lippman and Tanksley, 2001; Cong et al., 2008). The expression pattern expression of the *fas* gene is not altered in the *SlWUS* RNAi lines during floral development (**Figure 5B**), and we hypothesize that *SlWUS* function is required downstream of or parallel to *fas* function. Moreover, WUS positively regulates the expression of *AG* (Lenhard et al., 2001; Lohmann et al., 2001), and *AG* is critical in determining stamen and gynoecium identity (Yanofsky et al., 1990). It has been reported that *TAG1* silencing lines show defects in stamen and carpel development (Pan et al., 2010). In our *SlWUS* RNAi lines, the number of stamens and carpels decreased (**Figure 4D**). In addition, the expression of *TAG1* was significantly down-regulated (**Figure 5C**). Thus, these results suggest that a negative autoregulatory mechanism that involves *TAG1* and *SlWUS* in tomato is similar to that in *Arabidopsis*, and the number of carpels in tomato may be regulated through a pathway involving the *SlWUS* and *TAG1 g*enes. Other key components of the WUS signaling pathway are provided by the CLAVATA (CLV) proteins (Schoof et al., 2000; Clark, 2001; Lenhard and Laux, 2003). The WUS and CLV3 feedback loop is especially closely connected to the control of meristem size in Arabidopsis (Schoof et al., 2000). It is known that mutations in the *CLV* signaling pathway genes cause meristems to enlarge, which can lead to extra organs in the flowers and fruits. In addition, a regulatory change in *SlCLV3* underlies the *fas* mutant phenotype (Xu et al., 2015). In the *SlWUS* RNAi lines, the CLV-WUS feedback loop was also mildly affected. The expression level of *SlCLV3* was significantly down-regulated (**Figure 5D**). These results indicated that tomato domestication relied on changes in the interaction of the CLV-WUS feedback loop. YABBY expression is not altered in the RNAi-*SlWUS* lines, which is further evidence that *fas* is not encoded by YABBY (Xu et al., 2015).

## Conclusion

The results presented here demonstrate that the down-regulation of *SlWUS* on an *lc* mutant background affects fruit size via decreasing the number of locules. This finding suggests that *SlWUS* plays a role in tomato fruit size, and the identification of other transcription factors that interact with *SlWUS* is still required. The *SlWUS* RNAi lines produced in our study provide a suitable tool to better illustrate the molecular mechanisms by which *SlWUS* may control locule number. Furthermore, further functional studies are required to investigate how these two SNPs affect the molecular function of *SlWUS* in tomato locules using CRIPSR/Cas9 gene editing technology (Brooks et al., 2014; Doudna and Charpentier, 2014; Belhaj et al., 2015).

## Author Contributions

HL and MQ designed and carried out the experiments, analyzed the results, and wrote the manuscript. MS, YiL, YuL, TX, and YaL provided scientific advice, and revised the manuscript. TL conceived the research area, provided scientific advice, and supervised the project.

## Conflict of Interest Statement

The authors declare that the research was conducted in the absence of any commercial or financial relationships that could be construed as a potential conflict of interest.
