# Supplementary material for: Tomato Transcription Factor SlWUS Plays an Important Role in Tomato Flower and Locule Development
Source: Front Plant Sci. 2017 Mar 31;8:457. doi: 10.3389/fpls.2017.00457 (PMC5374213; doi:10.3389/fpls.2017.00457)
Supplement: Supplementary file 1 [file Data_Sheet_1.docx]

Supplementary Table 1

Real-time RT-PCR primers used to amplify gene-specific regions

| Category | Gene locus identity ^A^ | Primers sequences（5’-3’） |
| --- | --- | --- |
| *SlWUS* | Solyc02g083950 | Sense：CCAGCAACTTACCCTTTTCTTG  Antisense: TAAAGCAGAGTTACCCCTTTGG |
| *FW2.2* | Solyc02g090730 | Sense: GCTTAAGAACCGTGGCTTTG  Antisense: TGATAAGGGGGCATGGTAAC |
| *YABBY* | Solyc11g071810 | Sense: GTAAGATGTGGGCATTGTGC  Antisense: TGTACTGTTGCCTTTGCAGC |
| *TAG1* | Solyc02g071730 | Sense: CTTGATGCCAGGGAGTTCAT  Antisense: ATCGAATTGCTGAGGTGGAG |
| *SlCLV3* | Solyc11g071380 | Sense: AAAGGAAGTTGCTCCTGTGAA  Antisense: CCTCTTAGCTCCCAATCAGC |
| *Ubiquitin3* | Solyc01g056940 | Sense: CCAAGATCCAGGACAAGGAA  Antisense: AAATCAAACGCTGCTGGTCT |

^A^ Gene locus identity based on the ITAG release 2.3 official annotations on the SL2.40 genome built by the International Tomato Annotation Group (ITAG). The SOL Genomics Network (SGN) <http://solgenomics.net/>

**C**

**B**

**A**


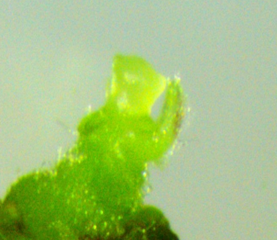

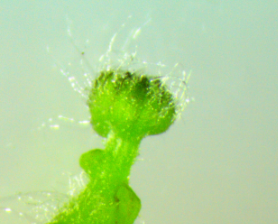

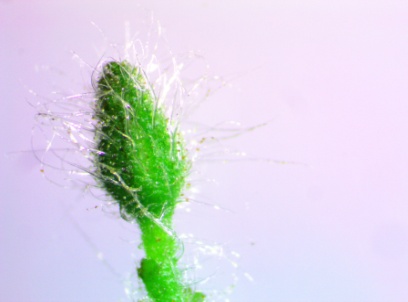


Supplementary Fig. 1 Sampling periods. A, Early development before the initiation of carpel primordial; B, Mid-stage development shortly after carpel initiation; and C, Late-stage development.
